# Comparisons of factors correlated with successful smoking cessation between middle-aged and older smokers

**DOI:** 10.1371/journal.pone.0342345

**Published:** 2026-02-10

**Authors:** Ya-Hui Chang, Jia-Ling Wu, Shang-Chi Lee, Chuan-Yu Chen, Chin-Wei Kuo, Esther Ching-Lan Lin, Shu-Ying Lo, Yu-Ying Huang, Chao-Ying Tsai, Pei-Hsing Hsieh, Chung-Yi Li

**Affiliations:** 1 Emergency Department, Shuang Ho Hospital, Taipei Medical University, New Taipei, Taiwan; 2 Graduate Institute of Injury Prevention and Control, College of Public Health, Taipei Medical University, Taipei, Taiwan; 3 Department of Public Health, College of Medicine, National Cheng Kung University, Tainan, Taiwan; 4 Department of Medical Research, E-Da Hospital, Kaohsiung, Taiwan; 5 Department of Family Medicine, National Cheng Kung University Hospital, College of Medicine, National Cheng Kung University, Tainan, Taiwan; 6 Division of Chest Medicine, Department of Internal Medicine, National Cheng Kung University Hospital, College of Medicine, National Cheng Kung University, Tainan, Taiwan; 7 Department of Nursing, College of Medicine, National Cheng Kung University, Tainan, Taiwan; 8 Department of Nursing, National Cheng Kung University Hospital, College of Medicine, National Cheng Kung University, Tainan, Taiwan; 9 Tobacco Control Division, Health Promotion Administration, Ministry of Health and Welfare, Taipei, Taiwan; 10 Department of Education and Humanities in Medicine, School of Medicine, College of Medicine, Taipei Medical University, Taipei, Taiwan; 11 Department of Public Health, College of Public Health, China Medical University, Taichung, Taiwan; 12 Department of Healthcare Administration, College of Medical and Health Science, Asia University, Taichung, Taiwan; University of Botswana School of Medicine, BOTSWANA

## Abstract

**Background:**

As the elderly population expands, smoking cessation becomes increasingly vital due to their heightened risk of adverse health effects. This study investigated the factors influencing successful smoking cessation among older smokers (≥65 years) in Taiwan, comparing them with those in middle-aged smokers (40–64 years).

**Methods:**

We conducted a retrospective cohort study involving participants aged 40 and above who engaged in smoking cessation therapies in Taiwan from 2012 to 2022. The primary outcome was self-reported smoking status six months post-treatment. Using logistic regression with generalized estimation equations, we calculated the adjusted odds ratios for successful cessation, considering various individual and ecological factors.

**Results:**

Among 263,641 middle-aged patients (442,133 treatment sessions) and 42,650 older smokers (67,372 treatment sessions), cessation success rates were 39.59% and 45.22%, respectively. Overall, both groups shared similar factors influencing cessation success. However, shorter smoking duration was more strongly associated with quitting among older smokers (adjusted odds ratio, aOR=1.35; 95% CI = 1.22–1.50), whereas varenicline (aOR=1.33; 95% CI = 1.26–1.40), bupropion (aOR=1.24; 95% CI = 1.19–1.30), and higher urbanization (aOR=1.29; 95% CI = 1.26–1.33) had greater effects among middle-aged smokers.

**Conclusions:**

While the underlying mechanisms of smoking cessation were similar across age groups, the relative influence of smoking duration, medication counseling, and institutional urbanization differed. These findings highlight the need for tailored interventions within the Tobacco Cessation Program, focusing on medication adherence and continuous counseling for middle-aged smokers, and improving accessibility and health management support for older adults.

## Introduction

Older adults who smoke are a key focus for tobacco cessation efforts due to their higher mortality rates compared to both younger smokers and older non-smokers or those who have quit [1]. Research shows that elderly individuals who stop smoking can significantly lower their risks of coronary heart disease, lung cancer, and chronic obstructive pulmonary disease [2]. However, studies indicate that older smokers often receive less support from healthcare providers for quitting, possibly due to concerns about managing treatments given age-related physiological changes and potential side effects [3,4]. Moreover, while numerous studies have investigated smoking cessation rates and influencing factors in smokers, there remains a shortage of research specifically targeting the elderly.

Phillips highlighted the necessity of customizing health education and cessation strategies to meet the unique needs of older smokers [5]. Nevertheless, it remains uncertain which strategies are most effective for this demographic or how varied factors might influence cessation success among different age groups within the older population. To effectively address the age-specific requirements of tobacco control, interventions must be appropriately tailored. It is important to note that resources, impacts, and cessation approaches can vary significantly across different age groups [6]. Additionally, they pointed out the distinction between preventative measures, which are more effective among youth, and the need for more resource-intensive and complex strategies to address late-stage addiction in older smokers. It is therefore essential to identify the factors most critical to successful cessation efforts among middle-aged and older smokers. Health professionals are called to deliver interventions specifically designed for older smokers.

Globally, smoking prevalence has declined in many high-income countries but remains substantial in low- and middle-income regions, particularly in the Western Pacific, where population aging and smoking overlap significantly [7]. Taiwan represents a distinctive case in this context. Since 2012, the government has implemented the Second-Generation Tobacco Cessation Program, a nationwide initiative designed to reduce financial barriers and broaden access to cessation services. The program allows smokers to receive up to two treatment courses per year, each combining pharmacotherapy and counseling, with substantially lowered copayments. It also extends service delivery beyond outpatient clinics to include inpatient, emergency, and pharmacy-based care, while providing subsidies for follow-up and case management to improve adherence and treatment quality [8]. This comprehensive system, integrated within Taiwan’s National Health Insurance, enables accessibility across socioeconomic groups and facilitates large-scale monitoring of cessation outcomes [9].

Understanding the determinants of cessation success within such a policy framework offers valuable insights for countries facing similar aging populations and health system challenges. Therefore, this study seeks to explore the factors contributing to successful smoking cessation among older smokers receiving services in Taiwan and compare the corresponding contributing factors for middle-aged smokers.

## Materials and methods

### Data source

We utilized the national Second-Generation Tobacco Cessation Program database, which was launched by the Health Promotion Administration in Taiwan on March 1, 2012. This program includes subsidized copayments for smoking cessation medications [10]. The eligibility criteria for individuals to receive pharmacotherapy for smoking cessation are as follows: (1) aged 18 years or older, (2) smoking more than 10 cigarettes a day or scoring 4 or higher on the Fagerström Test for Nicotine Dependence (FTND), and (3) seeking to quit tobacco smoking at an institution offering smoking cessation services [11]. Each treatment session allows for up to eight prescription refills within a 90-day supply. At the first clinic visit, participants provided demographic information and details on smoking-related behaviors, such as years of smoking and number of cigarettes smoked per day. The study was conducted with the approval of the Institutional Review Board of the National Cheng Kung University Governance Framework for Human Research Ethics (Approval No. 111–547). The requirement for a written informed consent form was waived because the data in our database was anonymous at the individual level. Data used in this study were obtained from the database and accessed on October 9, 2023.

### Study population

We initially identified 1,402,145 smoking cessation treatment sessions recorded between January 1, 2012, and December 31, 2022. After excluding sessions with missing identification numbers (n = 21,316), patients aged below 40 years (n = 517,331), and those lacking follow-up outcome information (n = 353,993), a total of 509,505 eligible sessions remained. We ultimately included 442,133 smoking cessation treatment sessions among 263,641 middle-aged patients and 67,372 treatment sessions among 42,650 older patients (S1 Fig).

### Six-months smoking cessation success

The participants self-reported their smoking status six months after the initial visit. To verify these self-reports, hospital staff and individual counselors conducted interviews either in person or over the phone to confirm the patients’ smoking status at six months post-treatment. The follow-up completion rate at this time was 59%.

### Covariates

At the initial treatment visit, each participant filled out a questionnaire that collected demographic information, number of smoking years, average number of cigarettes smoked per day, and nicotine dependence level (measured by the FTND). We categorized the medication therapies into three groups, either alone or in combination, including nicotine replacement therapy (NRT), varenicline, and bupropion. We also gathered data on the number of visits for the current treatment to categorize the level of utilization as high or low, and whether participants received health education counseling alone (yes or no). The classification of participants by their residential city district or township into categories of urbanization (high, medium, and low) was developed by Chang et al. [12], who divided a total of 358 city districts and townships in Taiwan into seven levels of urbanization using multiple demographic and socioeconomic indicators. In our study, these seven levels were further grouped into three categories—low (levels 5–7), medium (levels 3–4), and high urbanization (levels 1–2). The classification and data linkage were performed using SAS version 9.4. We also considered the type of institution where participants received treatment as a covariate to be adjusted in our study.

### Statistical method

Continuous variables are presented as the mean (standard deviation), and categorical variables are shown as the number (percentage). We examined differences in the six-month smoking cessation success rate between middle-aged and older smokers for each factor using logistic regression. Subgroup analyses were conducted for each factor to investigate the interactions between age and each variable stratum. For those factors where significant interactions were found, a logistic regression model incorporating a generalized estimating equation was employed to identify the adjusted odds ratios for individual and ecological factors significantly associated with the six-month smoking cessation success rate. We also calculated the percentage of explained variance (R-squared) to determine which factor explained the most variance in smoking cessation success.

## Results

Table 1 displays demographic characteristics for middle-aged and older smokers. Of those, 263,641 middle-aged patients received 442,133 treatment sessions, while 42,650 older patients accounted for 67,372 sessions. The average ages were 50.8 ± 6.9 for middle-aged and 71.0 ± 5.3 for older smokers. Older individuals had a higher proportion of males and tended to have smoked for longer durations with a medium to high FTND level.

**Table 1:** Characteristics of participants who received smoking cessation treatment.

	Middle aged smoker (n = 263,641)		Older smoker (n = 42,650)	
n (mean)	% (sd)	n (mean)	% (sd)
Treatment sessions^a^	442,133	100.0	67,372	100.0
Age (years)	(50.8)	(6.9)	(71.0)	(5.3)
Gender				
Male	358,132	81.0	59,335	88.1
Female	83,970	19.0	8,037	11.9
Season of first day of treatment session				
Spring	123,633	28.0	19,159	28.4
Summer	102,459	23.2	15,473	23.0
Autumn	97,204	22.0	14,749	21.9
Winter	118,837	26.9	17,991	26.7
Duration of smoking (year)				
<=10	26,141	5.9	1,806	2.7
11-20	103,639	23.4	4,035	6.0
21-30	186,920	42.3	8,678	12.9
31+	124,395	28.1	52,742	78.3
FTND				
Low (0–3)	24,769	5.6	4,400	6.5
Medium (4–6)	176,484	39.9	31,398	46.6
High (7+)	240,880	54.5	31,573	46.9
Medication^b^				
NRT	251,393	56.9	41,075	61.0
Varenicline	177,830	40.2	24,757	36.7
Bupropion	23,180	5.2	2,699	4.0
Along with health education counseling				
Yes	185,808	42.0	30,978	46.0
No	256,325	58.0	36,394	54.0
Urbanization of institution’s location				
High (1–2)	254,092	57.5	35,341	52.5
Medium (3–4)	136,626	30.9	20,667	30.7
Low (5–7)	51,415	11.6	11,364	16.9
Utilization^c^				
High	97,343	22.0	14,107	20.9
Low	344,790	78.0	53,265	79.1
Institution type				
Medical center	34,441	7.8	6,152	9.1
Regional hospital	73,917	16.7	11,129	16.5
Local hospital	45,792	10.4	7,417	11.0
Clinic	162,809	36.8	19,455	28.9
Public health center	47,438	10.7	11,860	17.6
Community pharmacy	77,736	17.6	11,359	16.9

FTND, Fagerström test for nicotine dependence; NRT, nicotine replace therapy; sd, standard deviation; Spring, March-May; Summer: June-August; Fall: September-November; Winter: December-February. ^a^Inconsistency between total population and population summed for individual variable was due to missing information

^b^One or more medication can be used in one treatment session.

^c^High utilization, received equal and more than 4 weeks of treatment during the session; Low utilization, less than 4 weeks of treatment during the session.

Middle-aged smokers achieved a six-month success rate in quitting smoking of 39.6%, while older smokers had a rate of 45.2%. Table 2 reveals the differences in success rates across various patient and institution characteristics, noting no significant difference with the use of bupropion (p = 0.2445). However, after adjusting for covariates, significant differences in smoking cessation success emerged between the two age groups, influenced by factors such as smoking duration, NRT, varenicline, and bupropion medications, health education counseling, and the urbanization level of the institution’s location.

**Table 2:** Overall and correlate-specific six-month smoking cessation success rate between middle-aged and older smokers.

	Middle aged smokers (n = 442,133)	Older smokers (n = 67,372)	P value for the difference in rate between middle-aged and older smokers	P-value for interaction^b^
	Number (%)	Number (%)		
Total^a^	175,021 (39.6)	30,465 (45.2)	<.0001	
Gender				
Male	142,300 (39.7)	26856 (45.3)	<.0001	0.4695
Female	32,706 (38.9)	3609 (44.9)	<.0001	
Season of first day of treatment session				
Spring	50,528 (40.9)	8812 (46.0)	<.0001	0.4741
Summer	39,975 (39.0)	7001 (45.2)	<.0001	
Autumn	37,154 (38.2)	6552 (44.4)	<.0001	
Winter	47,364 (39.9)	8100 (45.0)	<.0001	
Duration of smoking (year)				
<=10	12,059 (46.1)	957 (53.0)	<.0001	<.0001
11-20	41,513 (40.1)	1955 (48.5)	<.0001	
21-30	72,259 (38.7)	3965 (45.7)	<.0001	
31+	48,577 (39.1)	23523 (44.6)	<.0001	
FTND				
Low (0–3)	12,166 (49.1)	2331 (53.0)	<.0001	0.2685
Medium (4–6)	74,892 (42.4)	15069 (48.0)	<.0001	
High (7+)	87,963 (36.5)	13065 (41.4)	<.0001	
Medication				
NRT	96,192 (38.3)	18,240 (44.4)	<.0001	<.0001
Varenicline	74,542 (41.9)	11,752 (47.5)	<.0001	<.0001
Bupropion	8,153 (35.2)	953 (35.3)	0.2445	0.0002
Along with health education counseling				
Yes	80,601 (43.4)	15,332 (49.5)	<.0001	0.0034
No	94,420 (36.8)	15,133 (41.6)	<.0001	
Urbanization of institution’s location				
High (1–2)	103,546 (40.8)	16,279 (46.1)	<.0001	0.0008
Medium (3–4)	54,729 (40.1)	9,539 (46.2)	<.0001	
Low (5–7)	16,746 (32.6)	4,647 (40.9)	<.0001	
Utilization^c^				
High	40,764 (41.9)	6,745 (47.8)	<.0001	0.4670
Low	134,257 (38.9)	23,720 (44.5)	<.0001	
Institution type				
Medical center	15,278 (44.4)	3,172 (51.6)	<.0001	0.0602
Regional hospital	33,244 (45.0)	5,771 (51.9)	<.0001	
Local hospital	18,294 (40.0)	3,439 (46.4)	<.0001	
Clinic	53,838 (33.1)	6,984 (35.9)	<.0001	
Public health center	18,190 (38.3)	5,222 (44.0)	<.0001	
Community pharmacy	36,177 (46.5)	5,877 (51.7)	<.0001	

FTND, Fagerström test for nicotine dependence; NRT, nicotine replace therapy; Spring, March-May; Summer: June-August; Fall: September-November; Winter: December-February. ^a^Inconsistency between total population and population summed for individual variable was due to missing information

^b^The p-value for the interaction between age and each of the selected factor in multiple logistic regression with GEE.

^c^High utilization, received equal and more than 4 weeks of treatment during the session; Low utilization, less than 4 weeks of treatment during the session.

Separate regression analyses for middle-aged and older smokers are detailed in Table 3. We found that older smokers were more likely to quit if they had been smoking for less than 10 years (aOR=1.35, 95% CI = 1.22–1.50) and if they received medication treatment alongside health education counseling (aOR=1.10, 95% CI = 1.06–1.14). Conversely, middle-aged individuals benefited significantly from treatments involving varenicline (aOR=1.33, 95% CI = 1.26–1.40) and bupropion (aOR=1.24, 95% CI = 1.19–1.30), particularly in highly urbanized institution locations (aOR=1.29, 95% CI = 1.26–1.33).

**Table 3:** Covariate-adjusted odds ratios of successful smoking cessation between middle-aged and older smokers.

	Middle aged smokers	Older smokers	P-value for interaction^a^
	Adjusted Odds ratio (95% CI)	Adjusted Odds ratio (95% CI)	
Duration of smoking (year)			
<=10	1.24 (1.20-1.28)	1.35 (1.22-1.50)	<.0001
11-20	1.03 (1.01-1.05)	1.18 (1.10-1.27)	
21-30	0.98 (0.97-1.00)	1.08 (1.02-1.13)	
Medication			
NRT	1.02 (0.97-1.07)	1.05 (0.91-1.21)	<.0001
Varenicline	1.33 (1.26-1.40)	1.18 (1.02-1.37)	<.0001
Bupropion	1.24 (1.19-1.30)	1.03 (0.91-1.18)	0.0002
Along with health education counseling			
Yes	1.06 (1.04-1.07)	1.10 (1.06-1.14)	0.0034
Urbanization of institution’s location			
High (1–2)	1.29 (1.26-1.33)	1.12 (1.06-1.19)	0.0008
Medium (3–4)	1.23 (1.20-1.26)	1.13 (1.07-1.19)	

NRT, nicotine replace therapy.

^a^The p-value for the interaction between age and each of the selected factors in multiple logistic regression with GEE.

**Fig 1:**
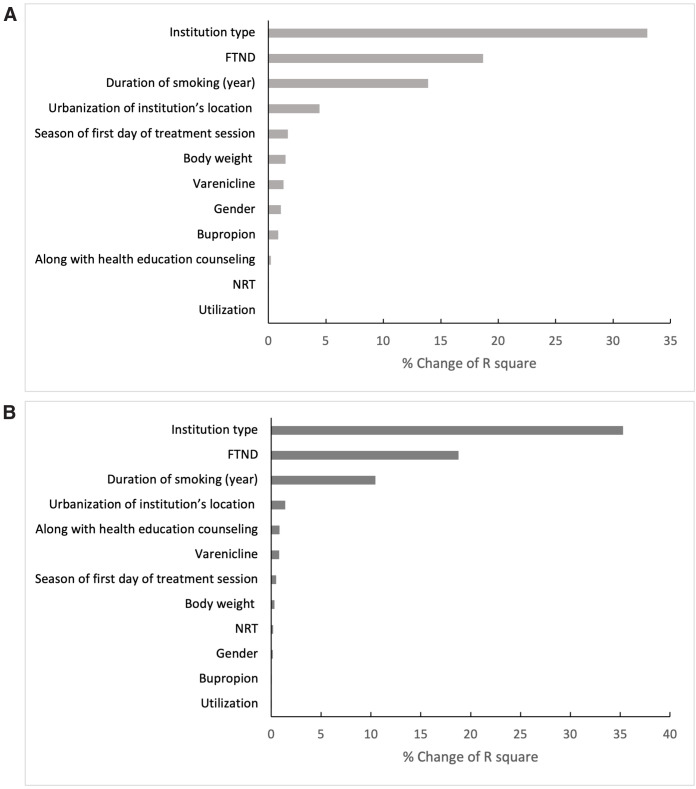
Degree of explanation of the associated between the factor and smoking cessation success. (A) Middle-aged adults. (B) Older adults.

Fig 1 illustrates how different variables explain variances in smoking cessation success. The type of institution, FTND levels, and the duration of smoking had a more significant impact than other factors, similarly observed in both middle-aged and older smokers. The combination of medication with health education counseling notably influenced the success of smoking cessation among older patients than in middle-aged smokers.

## Discussion

Middle-aged (ages 40–64) and older (ages 65+) smokers achieved six-month smoking cessation success rates of 39.6% and 45.2%, respectively. We found that older smokers were more likely to quit if they had smoked for less than 10 years and received medication combined with health education counseling. In contrast, middle-aged smokers had higher success rates with varenicline and bupropion medications, particularly when treated at institutions in highly urbanized areas. Better to compare the factors contributing to successful quitting between the two groups:

“Middle-aged and older smokers essentially shared similar factors that contributed to the success of smoking cessation, with shorter duration of smoking associated with more in older smokers than in middle-aged smokers. On the other hand, both varenicline and bupropion, as well as higher urbanization, were associated more with successful quitting in middle-aged than in older smokers.”

Our six-month smoking cessation success rate for older smokers was higher than the 35.1% rate reported in Turkey by Çetinkaya et al. for patients aged 60 and over who participated in a smoking cessation outpatient clinic program [13]. Our older smokers also showed a higher six-month smoking quit success rate than middle-aged smokers, which is consistent with previous studies [14,15]. For example, the six-month follow-up quit rates for smokers aged 45–64 were 33.2%, and 47.1% for those 65 and older in primary health care centers [14]. Older smokers may have a stronger motivation to quit smoking than middle-aged smokers, likely due to health concerns [16–18]. Health-related factors, such as taking more medications or having chronic conditions [17,18], were positively associated with smoking cessation in smokers aged 60 years or older. In addition, a newly diagnosed chronic health condition might appear to motivate older smokers to quit smoking but did not motivate middle-aged smokers [17].

Older smokers are more likely to quit if they have smoked for fewer years. This observation is supported by literature indicating that a shorter smoking duration increases the likelihood of successful cessation [19–21]. Additionally, studies have shown that individuals who start smoking at an older age have higher quit rates compared to those who begin at younger age [22,23]. Al-Dahshan et al found that individuals who began smoking after the age of 18 had a significantly higher quit rate than those who started at or before 18 years old [14], suggesting that an early onset of smoking complicates cessation efforts. However, Chang et al. found that among smokers aged 60 and above, the duration of smoking does not differ significantly between those who quit and those who continue [21].

The effectiveness of varenicline and bupropion was significantly different according to age groups [24]. Those findings were similar to our findings that varenicline and bupropion were more effective for successful smoking cessation in middle-aged than older smokers. Chang et al. proposed an explanation of the adverse effects of varenicline that may cause physicians to be more willing to prescribe varenicline to younger smokers than older smokers [25]. It is plausible that adherence to varenicline was poorer in older smokers, but this remains uncertain and warrants further investigation. Scholz et al. showed the success rates for older groups using NRT were equal to or better than those of older groups using bupropion and varenicline, which was also consistent with our present findings [24].

We also found that medication therapy, along with health education counseling, played a more significant role in contributing to smoking cessation among older smokers. The findings are consonant with those of Kim et al., who found that higher odds of smoking cessation for 6 months were positively associated with higher levels of behavioral counseling sessions, but this study did not investigate the age stratification for counseling association with quit rate [16]. Huang et al. also showed that effective counseling is an essential factor for successful smoking cessation [26]. Cohen-Mansfield et al. also observed that more social contact or support may be associated with smoking cessation for older people [18].

Smokers treated in urbanized areas appear to benefit more from quitting programs, particularly middle-aged smokers; however, this benefit was less obvious in older smokers. The urban clinics likely offer advantages due to superior resources and accessibility [27]. Nonetheless, these advantages might not be as effective for older smokers, potentially due to socioeconomic factors. Kim et al. noted that socioeconomic status (SES) inequalities in cessation rates increase among young and middle-aged individuals but begin to diminish after the age of 60 [16]. In contrast, data from the China Health and Retirement Longitudinal Study (CHARLS) suggest that the urban or rural location of residents was not significantly associated with smoking relapse [28].

The strengths of our study included the highlighting of differences in factors that affect smoking cessation between middle-aged and older smokers in a large, population-based sample in a clinical setting. We performed regression modeling to adjust for important confounding factors (including smoking years and type of medication) by separating the age-specific groups. In addition, our study was population-based, which covered all smokers who sought treatments from the Second-Generation Tobacco Cessation Program. The study sample was therefore considered representative. The present study has several limitations. In our study, first, we don’t consider the individual’s health condition since such information was not routinely collected in the national Second-Generation Tobacco Cessation Program. The omission of these data may have introduced unmeasured confounding, as older adults’ health status could affect both their motivation to quit and cessation success. Second, we don’t have socioeconomic status information that may have led to additional residual confounding, given that access to cessation resources and treatment affordability often differ by socioeconomic background.

This study suggests that while the basic mechanisms of smoking cessation are similar across different populations, the success of specific interventions may be influenced by the middle-aged or older context due to differences in duration of smoking, medication, health education counseling, and urbanization of the institution’s location. These findings imply that Taiwan’s Tobacco Cessation Program could further improve its impact by tailoring intervention strategies to meet the distinct needs of each age group. For example, emphasizing medication adherence and continuous counseling support for middle-aged smokers, while prioritizing accessibility and comorbidity management among older adults.

Future research should examine the long-term health outcomes of smoking cessation across age groups and incorporate socioeconomic indicators to identify disparities in cessation success. Such evidence would provide valuable guidance for optimizing cessation services and ensuring their effectiveness among both middle-aged and older populations.

## Supporting information

S1 Fig Flow diagram of eligible participants. (PDF)
