# Supplementary figures and images for: Comparisons of factors correlated with successful smoking cessation between middle-aged and older smokers
Source: PLoS One. 2026 Feb 10;21(2):e0342345. doi: 10.1371/journal.pone.0342345 (PMC12890095; doi:10.1371/journal.pone.0342345)

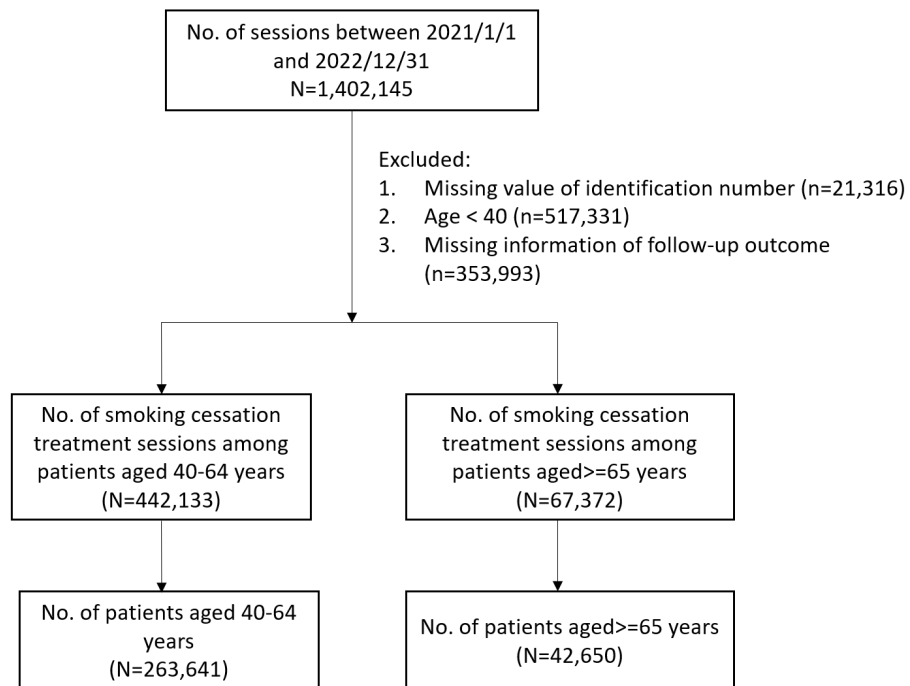

S1 Fig. Flow diagram of eligible participants

Supplement: S1 Fig — (PDF) [file pone.0342345.s001.pdf]
